# Stent placement in patients with acute subarachnoid haemorrhage: when is it justified?

**DOI:** 10.1007/s00234-018-2020-6

**Published:** 2018-04-11

**Authors:** Andrew G. Murchison, Victoria Young, Tanja Djurdjevic, Martino Cellerini, Rufus Corkill, Wilhelm Küker

**Affiliations:** 1 Department of Neuroradiology, John Radcliffe Hospital, Oxford University Hospitals NHS Foundation Trust, Oxford, UK; 2 Department of Neuroradiology, Medical University of Innsbruck, Innsbruck, Austria; 3 Nuffield Department of Clinical Neurosciences, University of Oxford, Headley Way, Oxford, OX3 9DU UK

**Keywords:** Aneurysm, Subarachnoid haemorrhage, Stenting

## Abstract

**Purpose:**

Endovascular stents are widely used for the elective treatment of cerebral aneurysms. Acute stenting is performed in the management of dissections, pseudo-aneurysms, broad-based aneurysms or as a ‘bail out’ measure after coil migration. The purpose of this study is to review the safety of using stents in acute subarachnoid haemorrhage.

**Methods:**

The stent registry of our institution was reviewed for procedures in patients with acute subarachnoid haemorrhage. Imaging studies were reviewed on the hospital’s PACS system and the patients’ notes were retrieved to assess complications and clinical outcomes. Procedures were analysed according to the type of stent, treatment indication, antiplatelet regime, complications and outcomes.

**Results:**

Between 2008 and 2016, 51 stents were placed during 50 stenting procedures in 49 patients with acute subarachnoid haemorrhage. This included 24 patients with saccular aneurysms, 10 with blister aneurysms, 10 dissections and five fusiform aneurysms. Stents were deployed in ‘bail out’ situations on eight occasions. In six cases, flow-diverting stents were used. Eighteen patients (37%) in the cohort suffered a stroke. Nine patients (18%) suffered persistent clinical deficits as a result of the stenting procedure, all but one of which occurred within 24 h. Two patients had a transient ischaemic episode, and there was evidence of asymptomatic ischaemia on imaging in four cases (8%). Five patients died, three (6%) as a result of procedural complications. Twelve patients (25%) required a further embolisation procedure.

**Conclusion:**

The use of stents in acute subarachnoid haemorrhage incurs a considerable complication risk and should be reserved for exceptional circumstances.

## Introduction

Coil embolisation has become the standard, evidence-based treatment for ruptured brain artery aneurysms since the landmark ISAT trial [1]. Subsequent technical developments have included balloon-assisted coiling for broad-based aneurysms [2] and endovascular stenting [3]. Balloon-assisted coiling is routinely performed in patients with ruptured aneurysms. However, endovascular stents are thrombogenic and require concomitant antiplatelet medication. This presents two problems in patients with acute intracranial bleeds; firstly, it increases the risk of bleeding complications, and secondly, the delayed onset of action of antiplatelet medication makes early stent thrombosis more likely. Therefore, stents are predominantly used for the treatment of unruptured aneurysms [4]. Despite the risks, certain situations may lead to the placement of a stent after subarachnoid haemorrhage; for example, if an aneurysm is too broad-based to safely contain a coil, if there is a vessel dissection or if there is the need to preserve flow when coil embolisation is complicated by coil migration or thrombus formation.

Adequate planning and patient consent require knowledge of the complication rates of acute stenting, especially where the use of a stent is considered prior to the procedure. It is also of great importance to determine the optimal antiplatelet medication for acute stenting and to establish the optimal device for this indication.

## Patients and methods

This study was conducted with appropriate institutional approval to audit against good practice and published treatment results. The prospectively kept central intracranial stent registry of the department was reviewed to identify all stenting procedures in patients with acute subarachnoid haemorrhage from 2008 to 2016. Acute stenting procedures were defined as those occurring within 30 days of presentation at hospital with acute ruptured subarachnoid haemorrhage. Relevant imaging studies were reviewed on the institutional PACS and the patients’ notes were retrieved to assess clinical outcomes.

All procedures were carried out under general anaesthetics by various operators. Aneurysm anatomy was assessed before the procedure by CTA according to the institutional standard protocol and/or by 3D DSA. For most patients, a standard 6F guide catheter was used. All patients received a bolus of heparin.

Due to the 9-year timespan of the review, various devices were used, reflecting the changing operator preferences and technical developments. As a result, a variety of delivery systems were also used: Prowler™ 21 for Enterprise™ (Codman Neuro, Raynham MA) and LVIS™ stents (Microvention Aliso Viejo CA), Vasco 21™ for Leo™ and SILK™ (BALT Extrusion, Montmorency, France), Marksman™ for Pipeline embolization device (PED) ™ (Medtronic, Minneapolis, MN), Excelsior SL10™ and Echelon 10™ (Stryker, Kalamazoo MI) for LVIS Jr. and Leo^+^ baby. Early Neuroform™ stents were preloaded on a dedicated delivery system. Later variants of the Neuroform stent were delivered through the Excelsior XT 27™, Rebar 27™ (Neuroform EZ™) or the Excelsior Sl 10™ (Neuroform Atlas™).

## Results

### Demographics and aneurysms

**Fig. 1:**
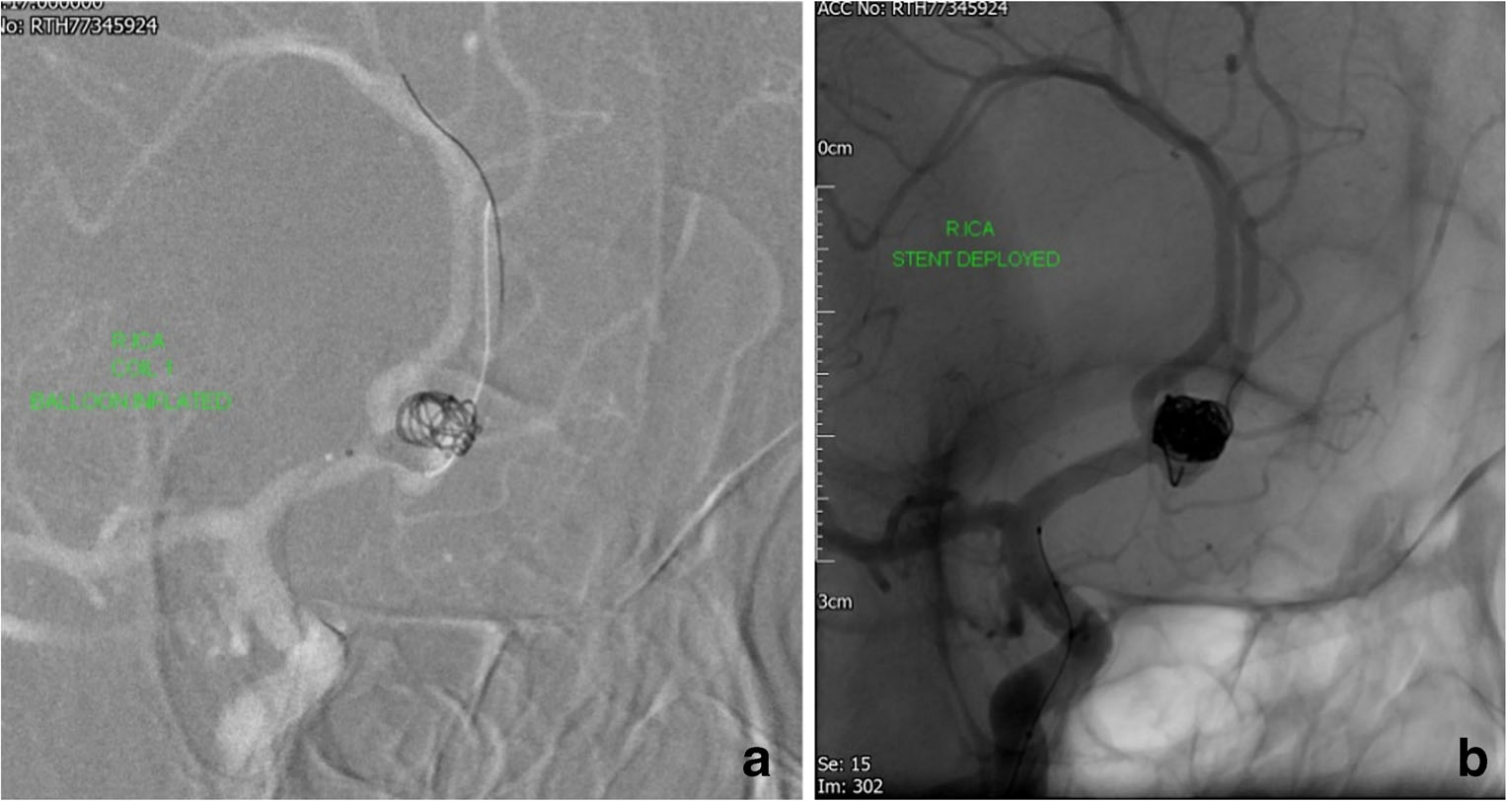
‘Bail out’ stenting during the treatment of a ruptured Acom aneurysm in a 63-year-old woman. There was coil loop migration into a A2 segment in spite of placement of a dual lumen balloon (Scepter XC) (**a**). A 2.5 × 18 mm Leo baby stent was deployed across the aneurysm neck without problems (**b**). Ten milligrams of ReoPro was given i.v. after deployment. The patient was placed on aspirin and clopidogrel from the next day. There were no ischaemic complications

**Fig. 2:**
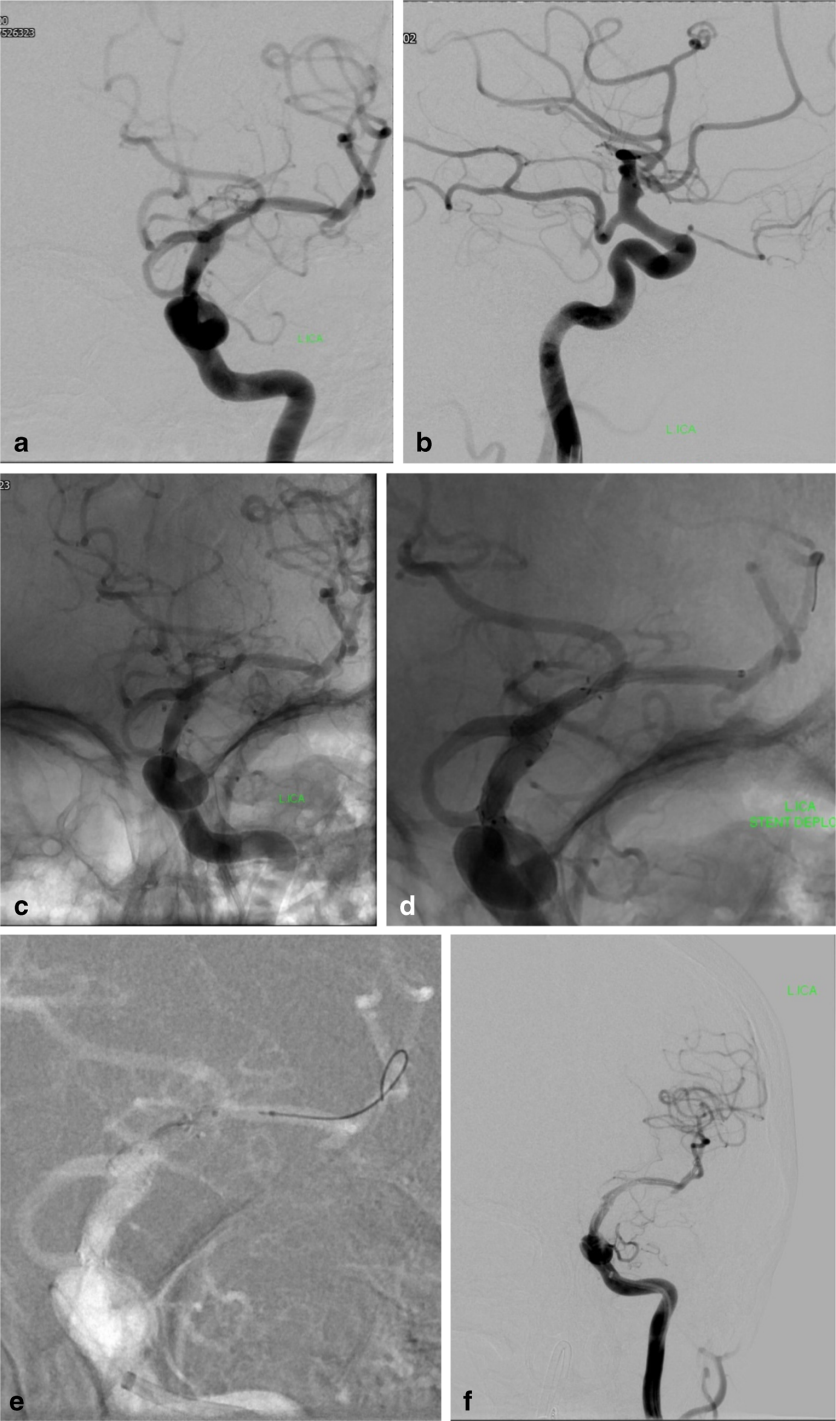
Treatment of a ruptured ‘blister’ aneurysm in a 68-year-old woman. The aneurysm is located above the origin of a fetal PCA (**a**, **b**). A Neuroform stent was deployed across the aneurysm and the PCA origin (**c**). It was not possible to telescope another Neuroform stent into the first one due to high friction of the microcatheter within the stent. Five hundred milligrams of aspirin was given i.v. after stent deployment. The patient was started on 75 mg of aspirin per day. The patient suffered a rebleed 20 days later. A Vasco microcatheter was advanced into the M1 segment across the Neuroform stent and a SILK flow-diverting stent was partially deployed in the M1 with the intention to pull the open distal end into the Neuroform stent (**d**). However, due to a significant distal stenosis of the Neuroform stent, which had not been appreciated, the SILK was constrained and fixated in this location (**d**). It was not possible to retrieve the SILK device. There was immediate clot formation with ICA occlusion. It was subsequently possible to pass the stenosis and perform an angioplasty with a Scepter balloon (**e**). A total of 20 mg of ReoPro were given i.v.. There was partial recanalization with persistent occlusion of some MCA branches. The transiently occluded ACA and PCA were filling from vertebral and contralateral ICA injections (not shown). In spite of only a small infarct, the patient failed to recover and died 1 week later

Fifty procedures in 49 patients fulfilled the selection criteria. Thirty-three women and 16 men (mean age 53 years) underwent stenting procedures after acute subarachnoid haemorrhage between 2008 and 2016 in our institution. One patient had a procedure involving the treatment of two separate aneurysms with stents, and one patient had two stenting procedures for the same aneurysm during the acute admission—therefore, a total of 51 stents were used to treat a total of 50 aneurysms. The underlying pathologies were saccular aneurysms in 24 patients, blister aneurysms in 10 (of which one patient had two blister aneurysms), dissections in 10 and fusiform aneurysm in five patients (Tables 1 and 2). Blister aneurysms were defined as broad-necked pseudo-aneurysms of the supraclinoid ICA. Eight procedures were carried out in ‘bailout’ situations because of coil migration (Fig. 1) with or without thrombus formation (*n* = 7) or acute vessel occlusion by a dissection (*n* = 1). One patient with a blister aneurysm was retreated after a rebleed from the aneurysm (Fig. 2).

**Table 1:** Aneurysms, treatment and outcomes by patient

Patient	Age	MRS at presentation	Days from presentation to treatment	Type of aneurysm (1 = berry, 2 = blister, 3 = dissection, 4 = fusiform	Location of aneurysm (1 = ICA, 2 = basilar, 3 = MCA, 4 = ACA, 5 = vertebral, 6 = PCA, 7 = PICA)	Size of aneurysm (mm)	Stent type (1 = LVIS, 2 = LEO, 3 = Neuroform, 4 = SILK, 5 = PED, 6 = Enterprise)	Dose of ReoPro (mg)	Loading dose of aspirin (mg)	Loading dose clopidogrel (mg)	Maintenance dose aspirin (mg)	Maintenance dose clopidogrel (mg)	Thrombus formation during procedure	Delayed retreatments (number)	Outcome (1 = no deficit, 2 = TIA, 3 = stroke)	Post-treatment MRS (6 = death)
1	39	1	1	1	3	2	1	0	500	0	75	75	No		1	0
2	40	1	0	1	4	8	1	10	500	0	0	75	Yes		3	4
3	58	1	0	1	4	6	1^•^	0	500	0	0	0	Yes		3*	6
4	64	5	1	1	4	7.3	2	15	500	300	75	75	Yes		3	2
5	39	1	1	1	4	6	2	10	500	75	75	75	No		1	0
6	63	1	5	1	4	2	2	0	500	0	75	75	No		1	0
7	71	1	27	1	2	2	2	0	500	0	75	75	No		1	0
8	56	1	1	1	2	14	3	0	500	0	0	75	No	1	1	0
9	64	2	1	1	1	4	3	0	500	0	75	75	No		1	0
10	45	1	0	1	2	16	3	0	500	0	75	75	No	2	3	0
11	60	1	11	1	4	3	3	0	500	0	75	75	No		1	6
12	42	1	1	1	1	2	3	0	500	300	75	75	No	1	1	0
13	44	5	0	1	4	9	3	20	500	300	75	75	No		1	6
14	36	1	4	1	1	3	5	0	500	0	75	75	No		1	0
15	62	1	1	1	2	11	6	0	500	300	75	150	No		1	0
16	52	4	7	1	4	12	6	0	500	300	150	150	Yes	1	1	2
17	62	1	1	1	3	2	6	0	500	600	75	75	No		2	0
18	62	5	0	1	4	2.5	6	20	0	0	0	75	No		1	3
19	67	2	2	1	1	10	6	0	500	300	75	75	No		1	4
20	48	1	12	1	4	1.5	6	0	500	0	75	75	No		1	0
21	64	1	1	1	4	4	6	0	500	0	0	75	No		1	2
22	35	4	1	1	4	8	6	20	500	0	0	75	No		1	1
23	80	1	0	1	4	14	6	0	500	300	75	75	No		2	0
24	72	5	4	1	1	6	2	0	500	0	75	75	No	1	1	2
25	51	1	1	2	1	2.5	1	0	500	0	75	75	No		1	1
26	44	1	1	2	1	2.2	3	0	500	300	75	75	No	3	3	4
27	63	1	1	2	1	2	3	0	500	0	75	75	No		1	0
27			1	2	1	3	6									
28	52	4	1	2	1	2	3	0	500	0	75	75	No		1	3
29	34	1	0	2	1	3	3	10	500	300	75	75	No	1	1	0
30	66	4	24	2	1	2	4	20	0	0	0	0	Yes	1	3*	6
31	42	4	1	2	1	5	5	0	500	0	75	75	No		1	0
32	63	4	1	2	1	2.3	6	0	500	300	75	75	No	1	1	1
33	34	5	4	2	1	2	6	0	500	0	75	75	No		1	1
34	43	1	2	2	1	4	4	20	500	300	75	75	Yes		1	2
35	52	5	0	3	5	0.6	1	0	500	300	75	75	No		1	4
36	43	2	2	3	4	6	2	10	500	300	75	75	Yes		1	0
37	52	5	1	3	5	4.5	2	10	500	600	75	75	Yes		3	0
38	25	1	5	3	2	1.6	6	0	0	0	75	75	No	2	1	2
39	39	1	2	3	5	11	6	0	0	0	75	75	No		1	0
40	56	1	1	3	2	6	6	0	500	0	NA	NA	No	1	1	1
41	22	1	1	3	3	16	6	0	0		0	0	No		3*	6
42	65	4	2	3	5	N/A	6	0	500	0	75	75	No		1	1
43	63	1	12	3	6	N/A	6	0	500	300	75	75	Yes		1	0
44	56	4	2	3	3	15	3	0	500	0	0	75	No		1	4
45	77	1	0	4	1	15	1	0	0	75	75	0	No		3	4
46	66	5	12	4	7	3	3	0	500	0	0	75	No		3	4
47	40	5	2	4	2	5	4	0	500	300	75	75	No		1	0
48	66	3	4	4	2	5	6^•^	0	500	300	150	150	No	4	1	2
49	66	5	2	4	7	4	5	0	500	180 ticagrelor	360 ticagrelor(180BD)	0	No		1	4

^•^In the stent type column denotes that the aneurysm was unsecured after the acute stenting treatment

*In the outcome column denotes a rebleed

**Table 2:** Summary of patient demographics and aneurysm characteristics

Patient demographics (*N* = 49)		
Age- median (range)		56 (22–80)
MRS at presentation- median (range)		1 (1–5)
Days from SAH to treatment- median (range)		3 (0–46)
Days from admission to treatment- median (range)		1 (0–27)
Aneurysm characteristics (*N* = 50)		
Aneurysm location- number (%)	ICA	17 (34)
	MCA	4 (8)
	ACA	14 (28)
	Basilar	8 (16)
	Vertebral	4 (8)
	PCA	1 (2)
	PICA	2 (4)
Aneurysm type- number (%)	Berry	24 (48)
	Blister	11 (22)
	Dissection	10 (20)
	Fusiform	5 (10)
Aneurysm size in mm- median (range)		4 (0.6–16)

### Procedural details and antiplatelet regimes

The most frequently utilised devices were Enterprise stents (*n* = 19), followed by Neuroform (*n* = 13), Leo (*n* = 7) and LVIS (*n* = 6). The flow-diverting stents SILK and PED were used in three procedures each (Tables 3, 4 and 5). The patient who had two stenting procedures was initially treated with a Neuroform stent, and with a SILK stent after a rebleed. Twenty-eight procedures entailed the placement of coils, with a range of 1 to 23 (median 4). Eight of 11 blister aneurysms were treated without additional coil placement and one coil was inserted to treat the remaining three aneurysms. External ventricular drains were placed prior to aneurysm treatment in 15 patients.

**Table 3:** Summary of stent types and outcomes

Stent type- number (%). *N* = 51	LVIS	6 (12)
	LEO	7 (14)
	Neuroform	13 (25)
	SILK	3 (6)
	PED	3 (6)
	Enterprise	19 (37)
Outcome- number (%). *N* = 49	No deficit	37 (76)
	TIA	2 (4)
	Stroke	10 (20)
MRS after treatment- median (range). *N* = 49		1 (0–6)

**Table 4:** Summary of the stents against the treated pathologies (*n* = 51 stents)

		Aneurysm				Total
		Berry	Blister	Dissection	Fusiform	
Stent	LVIS	3	1	1	1	6
	LEO	5	0	2	0	7
	Neuroform	6	5	1	1	13
	SILK	0	2	0	1	3
	PED	1	1	0	1	3
	Enterprise	9	3	6	1	19
Total		24	12	10	5	51

**Table 5:** Summary of the devices against outcomes (*n* = 49 patients)

		Outcome			Total
		No deficit	TIA	Stroke	
Stent	LVIS	4	0	2	6
	LEO	5	0	2	7
	Neuroform	9	0	3	12
	SILK	2	0	1	3
	PED	3	0	0	3
	Enterprise	15	2	1	18
Total		38	2	9	49

Antiplatelet medication varied. Five hundred milligrams aspirin was given in 44 procedures intravenously after stent implantation. Twenty patients also received clopidogrel, either immediately before or after the procedure (75 mg in two patients, 300 mg in 16 patients and 600 mg in two patients). One patient was given a loading dose of 180 mg ticagrelor™ (Astra Zeneca, Cambridge, UK).

The glycoprotein receptor IIb/IIIa antagonist abciximab (ReoPro™, Eli Lilly, Indianapolis IN) was used in 11 procedures to treat thrombus formation; 10 mg was given to five patients, 15 mg to one and 20 mg to five. Five thousand units of heparin were delivered at the time of the procedure in all cases; platelet function testing and post-operative heparisation were not routinely performed.

Forty-four patients were placed on a maintenance dose of aspirin (75 mg in 41 patients, 150 mg in three patients). Clopidogrel was prescribed in 37 patients (75 mg in 35 patients, 150 mg in two patients), and one patient was prescribed 180 mg ticagrelor twice per day (Table 1).

### Complications

Nine patients (18%) sustained new deficits following the procedure; ischaemic in seven and haemorrhagic in one patient (Tables 3 and 4). In the final patient, the stent was deployed in response to thrombus formation during coil embolisation, but the distal end of the stent failed to open. The combination of complications led to an infarct and subsequent a rebleed of the aneurysm. Acute stent thrombosis was noticed in four patients leading to ischaemic injury. In three cases, a clinical deficit was evident when the patient awoke after anaesthesia, and in the last case, the patient represented 2 years later with an infarct in the territory distal to the stent. All ischaemic strokes were in vascular territories downstream the embolised aneurysm, and none was watershed infarcts. In the case of the stroke with subsequent haemorrhage, the patient had already suffered a rebleed and vasospasm prior to the stenting. He then developed a massive increase in the extent of the haemorrhage during the procedure.

Two patients had a transient neurological deficit following the treatment. Four patients had ischaemia on neuroimaging without symptoms. Nine patients suffered strokes not immediately related to the stent placement. The causes were vasospasm (*n* = 5), thrombus formation during a coiling procedure for which a bailout stent was deployed (*n* = 1), an underlying dissection (*n* = 1), a previous coiling procedure (*n* = 1) and a subsequent embolisation procedure (*n* = 1).

Five patients died during hospital admission. Three deaths were procedure-related. The causes were rebleeding during the procedure and infarction due to stent thrombosis followed by a delayed rebleed in the second patient. The third patient died when a SILK flow-diverting became inverted and irretrievable during the retreatment of a blister aneurysm. This resulted in ICA occlusion with subsequent uncontrollable intracranial hypertension (Fig. 2). Two patients died because of complications related to the subarachnoid haemorrhage.

There were three rebleeds from the treated aneurysms, all in patients who subsequently died. There were no other intracranial haemorrhages due to the antiplatelet medication.

Rates of stroke (imaging and clinical) were comparable in patients who had bailout procedures (25%) and who had planned acute stenting procedures (19%). Three patients (13%) who had saccular aneurysms suffered strokes as a result of the stenting procedure, compared to two patients (20%) who had blister aneurysms. Treatment of dissections resulted in strokes twice (20%), as did the treatment of fusiform aneurysms (40%). Three patients (13%) with saccular aneurysms died, twice as a result of procedural complications. Mortality rates were 9% for blister aneurysms and 10% for dissections. No patient with a fusiform aneurysm died.

Thirty-one patients with acute subarachnoid haemorrhage were treated with laser-cut stents (Enterprise and Neuroform) of whom four (13%) suffered strokes and two had TIAs. Seven patients were placed on 75 mg of aspirin alone after placement of either an Enterprise or a Neuroform stent, of whom one suffered a stroke. The rest were placed on dual antiplatelet medication. Two patients (33%) suffered strokes after placement of the braided LVIS stent. One of these strokes resulted in the patient’s death. Three of 13 patients (23%) treated with either a flow-diverting stent (SILK and Pipeline) or the Leo braided stent suffered infarcts. Angiographic evidence of clot formation was present in six of these patients and was responsive to ReoPro injection in three cases. Four of seven patients treated with the Leo stent and two of three patients with SILK device were treated with ReoPro. ReoPro was only used in one of six LVIS procedures.

Patient numbers are too small for reliable statistical subgroup analysis. Table 5 summarises devices against procedure outcomes.

## Discussion

Spontaneous acute subarachnoid haemorrhage is a life-threatening condition. While the rupture of an intracranial saccular aneurysm is the most common underlying condition, other causes include dissections of cerebral arteries and fusiform aneurysms. The patients presented here comprise saccular aneurysms, very broad-based aneurysms, intracranial dissections and fusiform aneurysms.

Endovascular placement of platinum coils was shown to be superior to neurosurgical clipping in a prospective randomised trial several years ago [1]. Patients regarded as suitable for both treatment modalities were enrolled into the trial, which showed better functional outcomes and a marginally higher rebleeding rate after coiling. Although only a proportion of patients presenting with subarachnoid haemorrhage were randomised, endovascular treatment has widely replaced clipping as the standard of care for ruptured aneurysms. Coiling has also become the first-line treatment for vascular pathologies outside the ISAT trial, and for which there is no equally robust outcome data.

Endovascular treatment of aneurysms has been aided by subsequent technical developments such as balloons and stents [3]. While the use of balloon-assisted coiling for the treatment of ruptured broad-based saccular aneurysms is now regarded as standard of care [5], the use of stents in the acute situation remains problematic. Thromboembolic complications during balloon-assisted coiling can be reduced by peri-procedural heparin injection. In contrast, stents require platelet function inhibition until they are endothelialised. Depending on stent type, adequate levels of platelet inhibition may require the use of two synergistic drugs [3]. Aspirin, the most commonly used antiplatelet agent, can be injected intravenously and has an immediate onset of action. However, other commonly used drugs such as clopidogrel have a slow onset of action and cannot be administered intravenously (newer oral and intravenous antiplatelet agents were not available at the time of the treatments). For this reason, elective patients are given clopidogrel several days before the procedure, or several hours before the intervention as a loading dose. In acute subarachnoid haemorrhage, where this is not an option, stents are considered only if other, less risky treatment modalities are not available. Such situations include particularly adverse anatomy or an unforeseen procedural problem.

Fifty-one stents of varying types were implanted in 49 patients with acute subarachnoid haemorrhage in our institution during a 9-year period. In this time interval, a total of around 1400 acutely ruptured aneurysms were embolised and more than 300 intracranial stents were deployed (elective and acute cases). Owing to the retrospective nature of our review, and the time interval, a variety of devices were employed. Stent selection was based on device availability, operator experience and underlying vascular pathology. During the initial part of the study period, early versions of Neuroform stents prevailed, supplanted by Enterprise and later the Neuroform EZ and Neuroform Atlas. These devices were used for all indications including dissecting and blister aneurysms. Later, flow-diverting SILK and Pipeline devices were used, primarily in the setting of ICA blister aneurysms [6, 7]. The braided stents LVIS and Leo [8] were also used in this cohort. Until recently, LVIS Jr and LEO^+^ baby were the smallest stents available, and both are compatible with standard 0.017″ coiling microcatheters such as Excelsior SL10 and Echelon 10. This allowed the use of two microcatheters through one 6F guide catheter and ‘bail out’ stenting through a coiling catheter. Older stents require larger microcatheters (Prowler select 0.021″, Excelsior XT 27). Old versions of the Neuroform were preloaded on an ‘over the wire’ delivery system and had to be advanced over an exchange wire. This created additional problems in emergency situations.

Patients with blister aneurysms were treated with single or double stenting at the start of the study period, using conventional stents [9]. Later, these patients were treated with flow-diverting stents without additional coil placement [10, 11].

Braided stents and flow diverters are likely to be more thrombogenic than laser-cut stents. This may be reflected in a slightly higher stroke rate for the LVIS stents, the first braided stent used for acute stenting in our institution. The comparatively lower stroke rates for the Leo stents and flow diverters may reflect increasing experience in managing the antiplatelet medication for these devices.

Four of 13 patients treated with Neuroform and three of 19 of patients treated with Enterprise stents were placed on aspirin alone as maintenance treatment. There was one ischaemic event attributable to these procedures. However, the number of patients is too small to adopt routine single antiplatelet medication after placement of laser-cut stents. While there were two rebleeds from the treated aneurysms, we did not encounter other intracranial haemorrhages such as from the placement of ventricular catheters. A low risk of haemorrhage after application of antiplatelet medication in the setting of acute SAH is consistent with recent literature [12, 13].

Acute clot formation was observed during 11 procedures. Standard treatment was intra-arterial injection of the glycoprotein IIb/IIIa antagonist abciximab [14] which has been long established in our unit. This resulted in at least partial clot resolution in nine patients. Four patients suffered a stroke. There were no clinically significant bleeding events following Reopro administration. ReoPro was only used in one of six LVIS procedures despite a high stroke rate. This may reflect the developing experience with using braided stents, which later lead to more aggressive treatment of clot formation. Treatment with abciximab appears most effective if it is initiated early, upon first signs of clot formation [15]. Higher doses are required if a thrombotic stent occlusion has already occurred. Tirofiban has also been used for this purpose [16] and with similar results.

The death rate was 13% for patients with saccular aneurysms and 10% overall. This appears consistent with other case series of stenting in acute SAH [17, 18] [19] but is significantly higher than among all patients treated for ruptured aneurysms [3].

## Conclusion

Stenting in patients with acute subarachnoid haemorrhage carries a higher complication rate than coiling alone. For difficult aneurysms/dealing with complications, it may be appropriate and should be considered on an individual basis.
